# Percutaneous Coronary Intervention with Procedural Unfractionated Heparin without Activated Clotting Time Guidance: A Unique Opportunity to Assess Thrombotic and Bleeding Events

**DOI:** 10.1155/2024/6219301

**Published:** 2024-02-15

**Authors:** Ali Z. Zgheib, Jennifer Jdaidani, Elie Akl, Suzan Khalil, Omar Chaabo, Nicolo Piazza, Fadi J. Sawaya, Abdallah G. Rebeiz

**Affiliations:** ^1^American University of Beirut Medical Center, Division of Cardiology, Beirut, Lebanon; ^2^McGill University Health Centre, Division of Cardiology, Montreal, Canada; ^3^University of California at Los Angeles, Division of Cardiology, Los Angeles, California, USA

## Abstract

**Background:**

Rates of major bleeding and intraprocedural thrombotic events (IPTE) in the setting of percutaneous coronary intervention (PCI) using weight-adjusted unfractionated heparin (UFH) without activated clotting time (ACT) monitoring are not known.

**Methods:**

We reviewed 2,748 consecutive patients who underwent coronary angiography at our tertiary care university hospital between January 2017 and December 2020. All patients who underwent PCI with weight-adjusted UFH without ACT guidance were considered for further analysis. Major bleeding complications occurring within 48 hours of PCI were collected from patients' medical records. IPTE were collected independently by two interventional cardiologists after review of coronary angiograms.

**Results:**

There were 718 patients included in the analysis (65.4 ± 12.2 years old; 81.3% male). In total, 45 patients (7.8%) experienced a major bleed or IPTE. The most common IPTE were slow/no reflow (1.5%) and coronary artery dissection with decreased flow (1.1%). Other IPTE occurred in <1% of cases. Major bleeding occurred in 11 patients (1.5%), of whom 8 required blood transfusion and 3 required vascular intervention. Bleeding complications were more common with femoral compared with radial access (6.6% vs. 0.2%, *P* < 0.001).

**Conclusion:**

Weight-adjusted UFH use during PCI without ACT monitoring was related to low rates of major bleeding or IPTE.

## 1. Introduction

Unfractionated heparin (UFH) remains the most commonly used anticoagulant during percutaneous coronary interventions (PCIs) [1, 2]. UFH contains glycosaminoglycans which bind to antithrombin III, catalysing the inhibition of several coagulation factors, especially factors IIa and Xa [3]. However, UFH has an unpredictable therapeutic response because of marked variability in bioavailability, in part due to variable binding to plasma proteins and endothelial cells [4]. For this reason, the activated clotting time (ACT) test is used to monitor its therapeutic effect [5]. Current guidelines recommend target ACT values within 200 to 250 seconds with planned use of glycoprotein IIb/IIIa (GP IIb/IIIa) inhibitors and 250 to 300 seconds (HemoTec device) or 300 to 350 seconds (Hemochron device) in the absence of GP IIb/IIIa inhibitors [2].

Recently, the financial crisis in Lebanon generated significant medical equipment shortages, such that routine ACT testing became unavailable. Therefore, many PCI procedures were performed with unmonitored UFH at the American University of Beirut Medical Center. This provided a unique opportunity to assess thrombotic and bleeding events associated with this approach.

## 2. Methods

### 2.1. Study Design and Population

This is a single-center retrospective observational study conducted to assess intraprocedural thrombotic events (IPTE) and major bleeding complications during PCI performed with unmonitored UFH. We reviewed consecutive patients who underwent PCI with UFH for any indication between January 2017 and December 2020. All patients who received an alternative anticoagulant or whose UFH dosage was guided by ACT were excluded. Coronary angiography was performed according to validated standards. The choice of guiding catheter, guidewire, balloon, and stent size was decided by the interventional cardiologist. As per hospital protocol, patients undergoing PCI with UFH received the guideline-recommended weight-adjusted boluses of 70–100 U/kg or 50–70 U/kg with planned use GP IIb/IIIa inhibitor. There is no unified protocol for additional UFH boluses during prolonged PCI procedures at our institution. Patients diagnosed with out-of-hospitalST-segment elevation myocardial infarction (STEMI) were given a loading dose of 4000 units of UFH in the Emergency Department, followed by completion of the dose at the time of primary PCI (within 90–120 minutes) for a total of 70–100 U/kg. Hemostasis was achieved with a compression bracelet in case of radial access and manual compression in case of femoral access.

### 2.2. Statistical Analysis

Data management and analyses were carried out using SPSS Version 22.0 (IBM Corp, Armonk, New York). Descriptive statistics were performed by calculating the counts and percentages for categorical variables and mean and standard deviation for continuous variables. Categorical variables were compared using Pearson's chi-square test. A *P* value of 0.05 was used for significance.

### 2.3. Endpoints

IPTE were subcategorized into (1) no reflow or slow reflow; (2) coronary artery dissection with decreased coronary flow; (3) acute stent thrombosis (<24 hours); (4) persistent side branch compromise; (5) new or increasing coronary thrombus; and (6) abrupt vessel closure. Major bleeding was defined as bleeding requiring transfusion or vascular intervention within 48 hours of PCI.

### 2.4. Data Collection

Coronary angiograms, procedural reports, and medical records were reviewed retrospectively for prespecified clinical and procedural variables. Thrombotic and bleeding complications were validated independently by two interventional cardiologists. Study patients were assigned a unique identification number to ensure privacy of study data. The study was approved by the research ethics committee at our institution.

## 3. Results

### 3.1. Study Population and Procedural Characteristics

Out of 2,748 consecutively screened patients, 718 patients who underwent PCI without ACT monitoring were included in our study. Most patients were male (81.3%), and mean age was 65.4 years. Cardiovascular risk factors included hypertension (67.7%), dyslipidemia (53.6%), diabetes (42.8%), and active smoking (45.3%). Over a third of patients (36.2%) had a history of PCI, and 9.9% had a history of coronary artery bypass grafting (Table 1). Most procedures were done radially (78.3%), and the most common presentation was stable coronary artery disease (55.7%) (Table 2). The majority of patients (82.3%) received an ad hoc loading dose of an oral P2Y12 inhibitor at the time of PCI, and the most commonly prescribed agent was clopidogrel (69.5%). In total, 465 (64.8%) PCI procedures involved the left anterior descending artery, and none involved coronary bypass grafts. The number of implanted stents ranged from 0 to 7 (mean: 1.64; standard deviation: 0.9), with 0 representing unsuccessful PCI or conventional balloon angioplasty. Among the 87 patients presenting with STEMI, 33 received tirofiban at the discretion of the interventional cardiologist.

### 3.2. Outcomes

A total of 45 patients (6.3%) experienced at least one thrombotic or bleeding event (Table 3). The most frequently reported IPTE were slow/no reflow (*n* = 11, 1.5%) and coronary artery dissection with reduced flow (*n* = 8, 1.1%). Other IPTE occurred in less than 1% of cases and consisted of acute stent thrombosis (*n* = 5), persistent side branch compromise (*n* = 4), and new or increasing coronary thrombus (*n* = 2). There was no abrupt vessel closure.

Major bleeding requiring blood transfusion (*n* = 8) or vascular intervention (*n* = 3) within 48 hours of PCI occurred in 11 patients (1.5%). They occurred more frequently in patients undergoing PCI with femoral access compared with radial access (6.6% vs. 0.2%, *P* < 0.001).

Four patients did not survive to hospital discharge. One patient died during primary PCI of the left main coronary artery and had already sustained several cardiac arrests in the Emergency Department. The other three causes of death were listed as follows: (1) multiorgan failure, (2) severe anoxic brain injury after out-of-hospital cardiac arrest, and (3) mixed cardiogenic and septic shock.

## 4. Discussion

In this study, PCI performed with UFH was related to low rate of thrombotic and bleeding complications when no ACT guidance was used. To our knowledge, this is the first study to report the acute procedural outcomes of patients undergoing PCI with unmonitored UFH.

### 4.1. Evidence for ACT Testing

ACT testing at the time of PCI has been proposed to minimize the risk of periprocedural ischemic or bleeding events. However, despite its widespread use and guideline endorsement, results of various studies about this topic seem conflicting. In a meta-analysis of 6 randomized trials (*N* = 5,216 patients), ACT between 350 and 375 seconds provided the lowest composite ischemic event rate [6]. More recently, a Mayo Clinic study totalling 12,055 patients found no independent association between ACT values and in-hospital or 1-year ischemic or bleeding events [7]. Furthermore, in patients undergoing PCI with planned GPIs, no correlation was found between ACT and ischemic events in both the TAO [8] and FUTURA/OASIS-8 [9] trials. The occurrence of periprocedural bleeding and thrombotic events is likely multifactorial and difficult to predict. This is reflected in the 2018 European Society of Cardiology guidelines on myocardial revascularization in which no specific recommendation is made about the role of ACT testing in current PCI practice [10].

### 4.2. Radial vs. Femoral Access Site

In our analysis, radial access was associated with fewer bleeding complications compared with femoral access (0.2% vs. 6.6%, *P* < 0.001), which reflects prior data [11, 12]. In a meta-analysis of 9 randomized controlled trials totalling 10,760 patients with acute coronary syndrome, radial access was associated with decreased mortality (odds ratio (OR): 0.71; 95% confidence interval (CI): 0.56–0.90; *P*=0.004), major bleeding (OR: 0.55; 95% CI: 0.41–0.73; *P* < 0.001), and vascular access complications (OR: 0.32; 95% CI: 0.20–0.52; *P* < 0.001) at 30 days [13]. Furthermore, the ACT threshold predicting bleeding was higher for the radial (290 s) than the femoral approach (240 s) in the TAO trial [8]. Put together, these results can help adjust clinical decision making regarding the use of ACT testing and intensity of anticoagulation according to PCI access site, especially when resources are limited.

### 4.3. Comparison to Larger Databases

Although no direct comparison can be drawn, ischemic and bleeding event rates in our study are comparable to those reported in larger databases. In a contemporary ACS population of over 8,600 patients, rates of no/slow reflow (1.5%), dissection with decreased flow (1.2%), stent thrombosis (0.7%), and new procedural thrombus (0.2%) were almost numerically identical to those reported in our study (1.5%, 1.1%, 0.7%, and 0.3%, respectively) [14]. We observed a low rate of major bleeding of 1.5% in our study population. These rates are lower compared to larger studies [15], likely to due to varying definitions of major bleeding, transfusion thresholds, and follow-up durations.

### 4.4. Limitations

Our study was a single-center retrospective study with a modest sample size. Given that ACT testing remained available for select patients during the study period, UFH's therapeutic effect may have been monitored in complex cases based upon operator preference. This could explain the paucity of patients with bypass graft (0%) or left main (2.2%) PCI in our analysis; our results may thus not be applicable to higher risk populations. Furthermore, we did not compare outcomes with patients who received ACT monitoring. Clinical outcomes were not collected; however, none of the 4 reported mortalities were directly attributable to major bleeding or IPTE. Since GP IIb/IIIa inhibitors were used in less than 5% of cases in our study, caution should be exercised when extrapolating our findings to situations involving frequent GP IIb/IIIa inhibitor use. In addition, it is important to note that our hospital protocol, which excludes STEMI cases, necessitates the withholding of baseline anticoagulation for all patients. However, the retrospective nature of our study resulted in a lack of systematically available detailed information regarding baseline anticoagulant intake, including warfarin or DOACs; however, given the small percentage of STEMI patients in our cohort (12%), the impact of baseline anticoagulation on overall results is expected to be minimal. Finally, the restrictive transfusion protocol requiring a hemoglobin of 7.0 g/dL or less at our institution may have reduced the rate of reported major bleeding events.

## 5. Conclusion

In this single-center study, weight-adjusted UFH use during PCI without ACT monitoring was related to low rates of major bleeding or IPTE.

## Figures and Tables

**Table 1:** Patient demographics (*N* = 718).

	Mean (count)	Standard deviation (percentage)
Age (years)	65.4	12.2
Weight (kg)	82.6	15.0
LVEF (%)	52.4	11.0
Sex		
Male	584	81.3
Female	134	18.7
Hypertension	486	67.7
Dyslipidemia	385	53.6
Diabetes mellitus	307	42.7
Smoking		
Current smoker	325	45.3
Ex-smoker	127	17.7
Never	266	37.0
Previous PCI	260	36.2
Previous CABG	71	9.9

CABG: coronary artery bypass grafting; LVEF: left ventricular ejection fraction; PCI: percutaneous coronary intervention.

**Table 2:** Procedural characteristics.

	Mean (count)	Standard deviation (percentage)
Time of procedure (min)	69	33.4
Number of stents	1.6 (range = 0–7)	0.9
Diagnosis		
Stable angina/stable CAD	400	55.7
NSTEMI	108	15.0
STEMI	87	12.1
Staged PCI	73	10.2
Unstable angina	50	7.0
Access site		
Radial	562	78.3
Femoral	151	21.0
Ulnar	5	0.7
Antiplatelet		
Glycoprotein IIb/IIIa inhibitor (IV)	33	4.6
Clopidogrel (oral)	499	69.5
Ticagrelor (oral)	192	26.7
Prasugrel (oral)	27	3.8
Oral antiplatelets preload	127	17.7
Oral antiplatelets ad hoc load	591	82.3
Lesions intervened *n* = 1108		
LAD territory	498	69.4
RCA territory	294	40.9
LCX territory	264	36.8
Ramus intermedius	26	3.6
Left main	16	2.2
Coronary bypass graft	0	0

CAD: coronary artery disease; LAD: left anterior descending; LCX: left circumflex; NSTEMI: non-ST-segment elevation myocardial infarction; PCI: percutaneous coronary intervention; RCA: right coronary artery; STEMI: ST-segment elevation myocardial infarction.

**Table 3:** Outcomes.

	Count	Percentage
Total	45	6.3
IPTE		
No/slow reflow	11	1.5
Dissection with reduced flow	8	1.1
Stent thrombosis in <24 h	5	0.7
Side branch compromise	4	0.6
New/increased thrombus	2	0.3
Abrupt vessel closure	0	0
Major bleeding		
Bleeding requiring transfusion	Ulnar/radial access: 1 Femoral access: 7	0.2 4.6
Bleeding requiring vascular intervention	Ulnar/radial access: 0 Femoral access: 3	0 2.0
Total	Ulnar/radial access: 1 Femoral access: 10	0.2 6.6 *P* < 0.001

IPTE: intraprocedural thrombotic events.

## Data Availability

The data supporting the findings of this study are available upon request from the corresponding author.
